# Supplementary material for: Characterization of Parageobacillus Bacteriophage vB_PtoS_NIIg3.2—A Representative of a New Genus within Thermophilic Siphoviruses
Source: Int J Mol Sci. 2023 Sep 12;24(18):13980. doi: 10.3390/ijms241813980 (PMC10530707; doi:10.3390/ijms241813980)
Supplement: Supplementary file 1 [file ijms-24-13980-s001.zip › ijms-2569179-supplementary.pdf]

Supplementary material

Characterization of Parageobacillus Bacteriophage vB_PtoS_NIIg3.2—A Representative of a New Genus within Thermophilic Siphoviruses

Eugenijus Šimoliūnas [1,2,*], Monika Šimoliūnienė [1], Gintarė Laskevičiūtė [1], Kotryna Kvederavičiūtė [3], Martynas Skapas [4], Algirdas Kaupinis [5], Mindaugas Valius [5], Rolandas Meškys [1] and Nomeda Kuisienė [2]

[1] Department of Molecular Microbiology and Biotechnology, Institute of Biochemistry, Life Sciences Center, Vilnius University, Saulėtekio av. 7, LT-10257 Vilnius, Lithuania; monika.simoliuniene@gmc.vu.lt (M.Š.); gintare.laskeviciute@gmail.com (G.L.); rolandas.meskys@bchi.vu.lt (R.M.)

[2] Department of Microbiology and Biotechnology, Institute of Bioscience, Life Sciences Center, Vilnius University, Saulėtekio av. 7, LT-10257 Vilnius, Lithuania; nomeda.kuisiene@gf.vu.lt

[3] Department of Biological DNA Modification, Institute of Biotechnology, Life Sciences Center, Vilnius University, Saulėtekio av. 7, LT-10257 Vilnius, Lithuania; kotryna.kvederaviciute@mif.vu.lt

[4] Department of Characterisation of Materials Structure, Center for Physical Sciences and Technology, Sauletekio av. 3, LT-10257 Vilnius, Lithuania; martynas.skapas@ftmc.lt

[5] Proteomics Centre, Institute of Biochemistry, Life Sciences Center, Vilnius University, Saulėtekio av. 7, LT-10257 Vilnius, Lithuania; algirdas.kaupinis@gf.vu.lt (A.K.); mindaugas.valius@bchi.vu.lt (M.V.)

* Correspondence: eugenijus.simoliunas@bchi.vu.lt; Tel.: +370-6-507-0467

Table S1. Bacterial strains used in this study to determine the host range of bacteriophage NIIg3.2.

Strain	Sensitivity to NIIg3.2	Relevant characteristics	Source or reference
Aeribacillus pallidus DSM 3670^T	–	type strain	DSMZ
Aeribacillus pallidus strain SIII-6	–	environmental isolate	[1]
Aeribacillus pallidus strain PT-10	–	environmental isolate	[1]
Aeribacillus pallidus strain NIIg-6	–	environmental isolate	[1]
Aeribacillus pallidus strain NIIg-7	–	environmental isolate	[1]
Aeribacillus pallidus strain NIIg-5	–	environmental isolate	[1]
Aeribacillus pallidus strain PK-8	–	environmental isolate	[1]
Aeribacillus pallidus strain P-5	–	environmental isolate	[1]
Aeribacillus pallidus strain SIII-3	–	environmental isolate	[1]
Aeribacillus pallidus strain NIIg-8	–	environmental isolate	[1]
Brevibacillus borstelensis strain P8	–	environmental isolate	[1]
Brevibacillus borstelensis strain P-4	–	environmental isolate	[1]
Geobacillus lituanicus DSM 15325^T	–	type strain	DSMZ
Geobacillus stearothermophilus DSM 22^T	–	type strain	DSMZ
Geobacillus thermodenitrificans DSM 465^T	–	type strain	DSMZ
Geobacillus thermodenitrificans strain PK-6	–	environmental isolate	[1]
Geobacillus thermodenitrificans strain SIII-1	–	environmental isolate	[1]
Geobacillus thermodenitrificans strain NIIg-1	–	environmental isolate	[1]
Geobacillus thermodenitrificans strain PK-1-10	–	environmental isolate	[1]
Geobacillus thermodenitrificans strain PT-4	+	environmental isolate	[1]
Geobacillus thermodenitrificans strain NIIg-2	+	environmental isolate	[1]
Geobacillus thermodenitrificans strain NIIg-9	–	environmental isolate	[1]
Geobacillus thermodenitrificans strain PK-11	+	environmental isolate	[1]
Geobacillus thermodenitrificans strain P-1	–	environmental isolate	[1]
Geobacillus thermodenitrificans strain PK-2	–	environmental isolate	[1]
Geobacillus thermodenitrificans strain PT-9	–	environmental isolate	[1]
Geobacillus thermodenitrificans strain PT-5	–	environmental isolate	[1]
Geobacillus thermodenitrificans strain PT-3	–	environmental isolate	[1]
Geobacillus thermodenitrificans strain NIIg-11	–	environmental isolate	[1]
Geobacillus thermodenitrificans strain P-2	–	environmental isolate	[1]
Geobacillus thermodenitrificans strain PK-5	–	environmental isolate	[1]
Geobacillus thermodenitrificans strain NIIg-10	–	environmental isolate	[1]
Geobacillus thermodenitrificans strain PK-1	–	environmental isolate	[1]
Geobacillus thermodenitrificans strain PK-3	+	environmental isolate	[1]
Parageobacillus caldoxylosilyticus strain P-4-70	–	environmental isolate	[1]
Parageobacillus caldoxylosilyticus strain PK-7	–	environmental isolate	[1]
Parageobacillus caldoxylosilyticus strain PK-4	–	environmental isolate	[1]
Parageobacillus caldoxylosilyticus strain SIII-4	–	environmental isolate	[1]
Parageobacillus caldoxylosilyticus strain P-6	–	environmental isolate	[1]
Parageobacillus thermoglucosidasius strain P-3	–	environmental isolate	[1]
Parageobacillus thermoglucosidasius strain SIII-8	–	environmental isolate	[1]
Parageobacillus toebii strain NIIg-3	+*	environmental isolate	[1]
Peribacillus butanolivorans DSM 18929^T	–	type strain	DSMZ
Ureibacillus terrenus strain NIIg-4	–	environmental isolate	[1]
Ureibacillus thermosphaericus strain PK-2-65	–	environmental isolate	[1]
Ureibacillus thermosphaericus strain P1	–	environmental isolate	[1]

+ – bacterial strain sensitive to phage NIIg3.2
– – bacterial strain insensitive to phage NIIg3.2
***** – bacterial strain used to isolated phage NIIg3.2

Table S2. NIIg3.2 ORFs with homologues in other viruses or cellular organisms.

PT-9.1 ORF (position in genome)	Predicted function (protein length aa)	Significant match (protein length, aa)	Identity/ similarity, % (length of the overlapping segment, aa)	E value
ORF01<br>(22 -> 516)	terminase small subunit (164)	UYL94096.1 terminase small subunit<br>*Geobacillus* phage vB_GthS_PK5.1 (166)	72/84 (165)	5e-77
ORF02<br>(513 -> 2,240)	terminase large subunit (575)	UYL94201.1 terminase large subunit<br>*Geobacillus* phage vB_GthS_PT9.1 (567)	65/79 (574)	0.0
ORF03<br>(2,212 -> 2,430)	hypothetical protein (72)	WP_044736331.1 hypothetical protein<br>*Geobacillus kaustophilus* (61)	90/91 (61)	2e-31
ORF04<br>(2,447 -> 3,748)	portal protein (433)	YP_512313.1 portal protein<br>*Bacillus* phage Fah (429)	69/84 (421)	0.0
ORF05<br>(3,672 -> 4,301)	prohead protease (209)	QOQ37244.1 prohead protease<br>*Bacillus* phage z1a (206)	75/86 (204)	3e-107
ORF06<br>(4,346 -> 5,548)	major capsid protein (400)	YP_010080158.1 major head protein<br>*Paenibacillus* phage Dragolir (391)	56/73 (398)	6e-154
ORF07<br>(5,561 -> 5,869)	hypothetical protein (102)	UGO51726.1 hypothetical protein<br>*Bacillus* phage vB_BanS_Athena (85)	37/53 (95)	8e-08
ORF08<br>(5,901 -> 6,185)	head-tail connector protein (94)	YP_001285813.1 head-tail connector protein<br>*Geobacillus* virus E2 (95)	67/86 (94)	8e-46
ORF09<br>(6,182 -> 6,502)	head closure protein (106)	YP_009193821.1 head closure Hc1<br>*Paenibacillus* phage Harrison (106)	33/51 (106)	6e-12
ORF10<br>(6,502 -> 6,888)	putative tail-component (128)	NP_690765.1 tail completion or Neck1 protein<br>*Bacillus* phage phi105 (127)	36/60 (121)	2e-19
ORF11<br>(6,885 -> 7,235)	putative tail-component (116)	QBP06944.1 DUF3168 domain-containing protein<br>*Virgibacillus* phage Mimir87 (130)	31/56 (81)	7e-04
ORF12<br>(7,239 -> 7,814)	major tail protein (191)	QIW89305.1 hypothetical protein<br>*Bacillus* phage vB_Bacillus_1020A (189)	46/64 (156)	8e-38
ORF13<br>(7,869 -> 8,213)	hypothetical protein (114)	YP_010739970.1 hypothetical protein<br>*Bacillus* phage z1a (105)	40/62 (72)	9e-07
ORF14<br>(8,210 -> 8,404)	hypothetical protein (64)	WP_061579702.1 hypothetical protein<br>*Parageobacillus caldoxylosilyticus* (64)	97/98 (64)	1e-36
ORF15<br>(8,422 -> 12,666)	tape measure protein (1414)	YP_009218077.1 tail tape measure protein<br>*Clostridium* phage phiCT19406C (1148)	33/54 (953)	4e-140
ORF16<br>(12,647 -> 14,101)	tail hub protein (484)	UYL94008.1 distal tail protein<br>*Geobacillus* phage vB_GthS_PK3.6 (477)	48/63 (480)	1e-142
ORF17<br>(14,110 -> 16,743)	tail fiber protein (877)	UYL93929.1 putative tail fiber protein<br>*Geobacillus* phage vB_GthS_PK3.5 (959)	52/65 (785)	0.0
ORF18<br>(16,706 -> 17,050)	hypothetical protein (114)	SFA50906.1 hypothetical protein<br>*Parageobacillus thermantarcticus* (114)	96/98 (113)	1e-64
ORF19<br>(17,091 -> 17,282)	hypothetical protein (63)	UYL93985.1 hypothetical protein<br>*Geobacillus* phage vB_GthS_PK3.5 (62)	85/98 (61)	6e-30
ORF20<br>(17,292 -> 17,537)	holin (81)	UYL94124.1 hemolysin<br>*Geobacillus* phage vB_GthS_PK5.1 (72)	67/77 (79)	4e-23
ORF21<br>(17,539 -> 17,760)	holin (73)	YP_009099315.1 holin<br>*Bacillus* phage Waukesha92 (76)	69/83 (71)	5e-28
ORF22<br>(17,778 -> 18,476)	N-acetylmuramoyl-L-alanine amidase (232)	YP_001285830.1 N-acetylmuramoyl-L-alanine amidase<br>*Geobacillus* virus E2 (233)	80/88 (233)	1e-129
ORF23<br>(18,469 -> 18,735)	hypothetical protein (88)	YP_764495.1 hypothetical protein<br>*Geobacillus* phage GBSV1 (70)	84/93 (64)	3e-31

ORF24 (18,780 <- 19,529)	hypothetical protein (249)	YP_001285831.1 hypothetical protein *Geobacillus* virus E2 (266)	31/53 (147)	2e-11
ORF25 (19,815 <- 21,299)	integrase (494)	YP_007010946.1 integrase Deep-sea thermophilic phage D6E (480)	72/87 (480)	0.0
ORF26 (21,331 <- 21,999)	transcriptional repressor (222)	YP_001285833.1 transcriptional repressor *Geobacillus* virus E2 (229)	65/80 (224)	e-96
ORF27 (22,199 -> 22,417)	XRE family transcriptional regulator (72)	QBP06976.1 helix-turn-helix domain-containing protein *Virgibacillus* phage Mimir87 (78)	63/77 (68)	3e-25
ORF28 (22,422 -> 22,559)	hypothetical protein (45)	UYL94193.1 hypothetical protein *Geobacillus* phage vB_GthS_PK5.2 (48)	72/84 (23)	0.002
ORF29 (22,556 -> 22,879)	hypothetical protein (107)	YP_001285836.1 hypothetical protein *Geobacillus* virus E2 (59)	73/84 (59)	2e-25
ORF30 (22,921 -> 23,163)	hypothetical protein (80)	NP_835524.1 hypothetical protein *Staphylococcus* phage phiN315 (71)	43/61 (75)	3e-13
ORF31 (23,236 -> 23,352)	hypothetical protein (38)	No relative homologues found		
ORF32 (23,517 -> 24,260)	Rha family transcriptional regulator (247)	UYL94087.1 Rha family transcriptional regulator *Geobacillus* phage vB_GthS_PK5.1 (242)	78/85 (157)	3e-129
ORF33 (24,257 -> 24,517)	hypothetical protein (86)	YP_007010950.1 hypothetical protein Deep-sea thermophilic phage D6E (96)	63/83 (84)	7e-36
ORF34 (24,514 -> 24,861)	hypothetical protein (115)	YP_009199192.1 hypothetical protein *Brevibacillus* phage Jenst (112)	56/75 (61)	1e-12
ORF35 (24,858 -> 25,022)	hypothetical protein (54)	ANT40099.1 hypothetical protein *Bacillus* phage vB_BtS_BMBtp15 (50)	50/71 (42)	4e-08
ORF36 (25,127 -> 25,399)	hypothetical protein (90)	YP_009216655.1 hypothetical protein *Clostridium* phage phiCT453A (143)	44/68 (75)	8e-17
ORF37 (25,413 -> 25,604)	hypothetical protein (63)	UTQ79995.1 hypothetical protein *Bacillus* phage BC-1 (88)	36/49 (67)	0.033
ORF38 (25,624 -> 25,899)	hypothetical protein (91)	PUF85784.1 hypothetical protein *Geobacillus* sp. LYN3 (62)	97/96 (62)	3e-35
ORF39 (25,896 -> 26,375)	Mu Gam-like end protection protein (159)	YP_001285841.1 Mu Gam-like end protection *Geobacillus* virus E2 (195)	96/98 (159)	4e-93
ORF40 (26,372 -> 27,199)	hypothetical protein (275)	YP_001285842.1 hypothetical protein *Geobacillus* virus E2 (266)	85/89 (275)	4e-165
ORF41 (27,211 -> 27,372)	hypothetical protein (53)	YP_007010954.1 hypothetical protein Deep-sea thermophilic phage D6E (71)	51/71 (53)	3e-13
ORF42 (27,377 -> 28,171)	DNA replication initiation protein DnaD (264)	AUG88590.1 DnaD *Bacillus* phage BVE2 (243)	46/63 (251)	1e-58
ORF43 (28,059 -> 28,955)	DNA replication protein DnaC (298)	YP_008240345.1 DnaC-like helicase loader *Thermus* phage phi OH2 (280)	88/94 (280)	0.0
ORF44 (28,962 -> 29,150)	hypothetical protein (62)	UYL94182.1 hypothetical protein *Geobacillus* phage vB_GthS_PK5.2 (67)	66/81 (58)	1e-17
ORF45 (29,150 -> 29,317)	hypothetical protein (55)	YP_008240343.1 hypothetical protein *Thermus* phage phi OH2 (58)	98/100 (54)	4e-31
ORF46 (29,440 -> 29,583)	hypothetical protein (47)	WP_155121009.1 hypothetical protein *Geobacillus thermodenitrificans* (47)	100/100 (47)	4e-24
ORF47 (29,580 -> 30,215)	dUTP diphosphatase (211)	YP_008240342.1 nucleoside triphosphate pyrophosphohydrolase *Thermus* phage phi OH2 (169)	57/66 (212)	2e-73
ORF48 (30,226 -> 30,363)	hypothetical protein (45)	KQB91905.1 hypothetical protein *Geobacillus* sp. PA-3 (117)	57/71 (42)	3e-07

ORF49<br>(30,360 -> 30,524)	hypothetical protein (54)	UYL93994.1 hypothetical protein *Geobacillus* phage vB_GthS_PK3.5 (54)	83/90 (54)	1e-18
ORF50<br>(30,534 -> 30,929)	YopX family protein (131)	YP_009223844.1 hypothetical protein *Geobacillus* virus E3 (126)	62/74 (129)	4e-47
ORF51<br>(30,926 -> 31,219)	hypothetical protein (97)	PUF85776.1 hypothetical protein *Geobacillus* sp. LYN3 (100)	89/95 (97)	3e-43
ORF52<br>(31,216 -> 31,518)	Holliday junction resolvase (100)	YP_001285859.1 hypothetical protein *Geobacillus* virus E2 (96)	92/97 (96)	3e-58
ORF53<br>(31,515 -> 31,895)	hypothetical protein (127)	WP_090952020.1 hypothetical protein *Parageobacillus thermantarcticus* (120)	87/91 (119)	1e-70
ORF54<br>(31,888 -> 32,256)	hypothetical protein (122)	YP_009223823.1 hypothetical protein *Geobacillus* virus E3 (114)	36/54 (124)	7e-15
ORF55<br>(32,272 -> 32,838)	hypothetical protein (188)	UYL94155.1 hypothetical protein *Geobacillus* phage vB_GthS_PK5.2 (201)	65/73 (205)	9e-78
ORF56<br>(32,848 -> 32,991)	hypothetical protein (47)	UYL94197.1 hypothetical protein *Geobacillus* phage vB_GthS_PK5.2 (45)	75/84 (44)	1e-11
ORF57<br>(33,031 -> 33,240)	hypothetical protein (69)	OQP00874.1 hypothetical protein *Geobacillus* sp. 44C (69)	97/98 (69)	3e-39
ORF58<br>(33,280 -> 33,411)	hypothetical protein (43)	KQB91902.1 hypothetical protein *Geobacillus* sp. PA-3 (43)	100/100 (43)	6e-21
ORF59<br>(33,457 -> 33,705)	hypothetical protein (82)	UYL94174.1 hypothetical protein *Geobacillus* phage vB_GthS_PK5.2 (90)	71/80 (85)	8e-33
ORF60<br>(33,702 -> 33,950)	hypothetical protein (82)	WP_183253994.1 hypothetical protein *Anoxybacillus tepidamans* (82)	79/89 (78)	4e-41
ORF61<br>(33,956 -> 34,468)	hypothetical protein (170)	YP_009206347.1 hypothetical protein *Bacillus* phage phi4B1 (154)	42/59 (127)	4e-30
ORF62<br>(34,483 -> 34,776)	hypothetical protein (97)	WP_060476214.1 hypothetical protein *Geobacillus* sp. PA-3 (119)	44/55 (109)	1e-12
ORF63<br>(34,773 -> 34,913)	hypothetical protein (46)	UYL94196.1 hypothetical protein *Geobacillus* phage vB_GthS_PK5.2 (46)	84/93 (45)	1e-07
ORF64<br>(34,773 -> 34,913)	transcriptional activator (151)	YP_008240337.1 transcriptional activator *Thermus* phage phi OH2 (150)	95/95 (150)	8e-100
ORF65<br>(35,361 -> 36,092)	hypothetical protein (243)	YP_009837596.1 hypothetical protein *Anoxybacillus* phage A403 (175)	42/56 (124)	7e-12
ORF66<br>(36,142 -> 36,594)	putative regulatory protein (150)	YP_001285865.1 excisionase *Geobacillus* virus E2 (171)	72/79 (68)	6e-26
ORF67<br>(36,587 -> 37,348)	hypothetical protein (253)	YP_009223742.1 hypothetical protein *Geobacillus* virus E3 (512)	25/49 (130)	6e-05
ORF68<br>(37,537 -> 37,698)	hypothetical protein (53)	YP_001504370.1 hypothetical protein *Geobacillus* phage GBSV1 (53)	74/86 (53)	6e-22
ORF69<br>(37,747 -> 38,166)	hypothetical protein (139)	WP_253922572.1 hypothetical protein *Halobacillus* sp. A5 (90)	53/71 (85)	5e-22
ORF70<br>(38,203 -> 38,427)	hypothetical protein (74)	MBY6269331.1 hypothetical protein *Parageobacillus thermoglucosidasius* (92)	67/69 (92)	2e-32
ORF71<br>(38,474 -> 38,884)	HNH endonuclease (136)	UYL93958.1 HNH endonuclease *Geobacillus* phage vB_GthS_PK3.5 (130)	58/79 (130)	9e-47

Table S3. NIIg3.2 structural proteins identified by Mass Spectrometry.

Gene	Putative protein function	MW (KDa)	Peptide count	Sequence coverage (%)
ORF15	tape measure protein	153.014	49	40.66
ORF17	tail fiber protein	95.973	9	13.45
ORF16	tail hub protein	54.884	6	16.32
ORF06	major capsid protein	45.625	19	44.75
ORF05	prohead protease	24.216	5	35.71

Table S4. Top BLASTp matches of gp17 from NIIg3.2 to viral proteins in the NCBI database.

Significant match (protein length (aa))	Length of the overlapping segment (aa)	Identity/ similarity (%)	E-value
UYL93929.1 putative tail fiber protein, *Geobacillus* phage vB_GthS_PK3.5 (959)	785	52/65	0.0
UYL94138.1 tail fiber protein, *Geobacillus* phage vB_GthS_PK5.2 (1316)	447	67/79	0.0
UYL94005.1 putative tail fiber protein, *Geobacillus* phage vB_GthS_PK3.6 (1307)	781	46/60	0.0
UYL94199.1 tail fiber protein, Geobacillus phage vB_GthS_PT9.1 (1237)	447	64/77	0.0
UYL93777.1 tail fiber protein, *Geobacillus* phage vB_GthS_NIIg9.7 (1237)	447	63/77	0.0

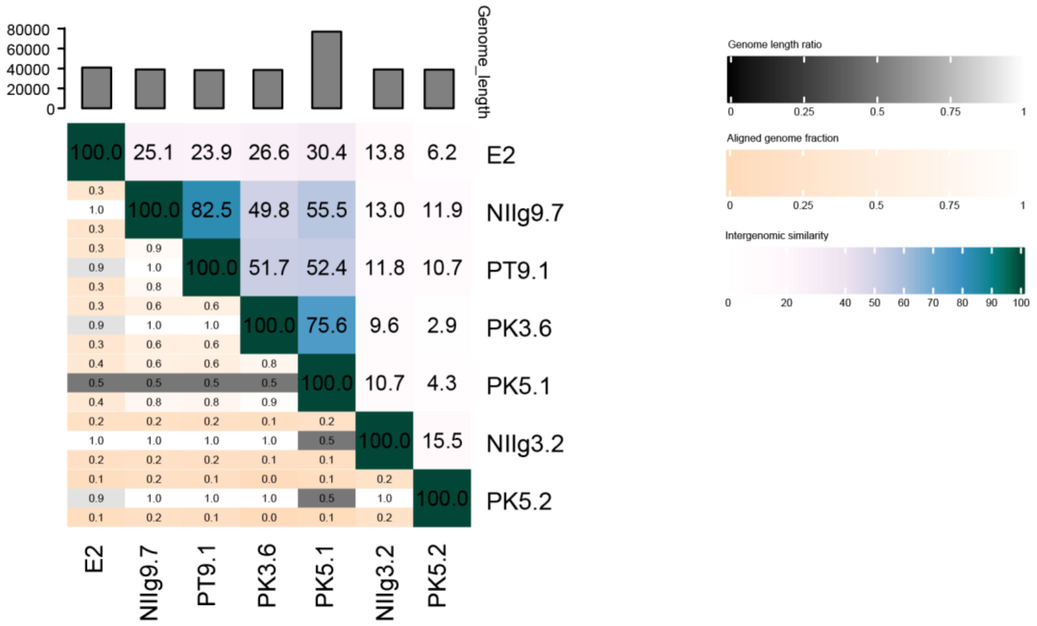

Figure S1. The whole-genome comparison and clustering of *Parageobacillus* phage NIIg3.2 and its closest relatives. The comparison and clustering were performed with the use of VIRIDIC. Different shades of blue in the right half of the heatmap represent different intergenomic similarities (%) between the genomes of each pair compared, as indicated above the heatmap and specified by numbers. The left half of the heatmap shows three indicator values for each genome pair: aligned fraction of genome one for the genome in this row (top value), genome length ratio for the two genomes in this pair (middle value) and aligned fraction of genome two for the genome in this column (bottom value).

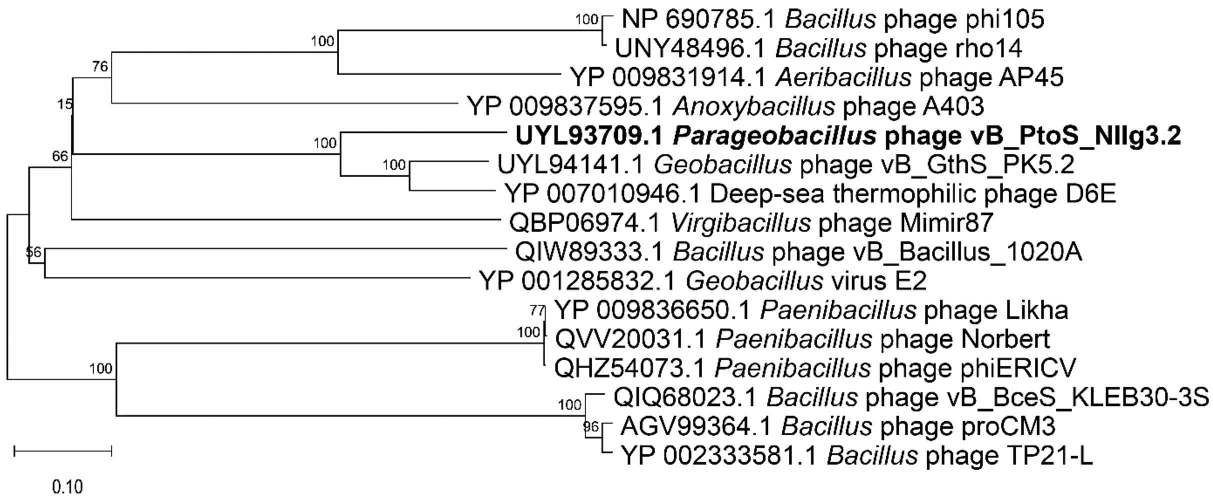

Figure S2. Neighbor-joining tree analysis based on the alignment of the amino acid sequences of *Parageobacillus* bacteriophage NIIg3.2 integrase (gp25) and its closest BLASTp viral homologues. The phylogenetic analysis was conducted using MEGA version 5. The percentage of replicate trees in which the associated taxa clustered together in the bootstrap test is shown next to the branches.

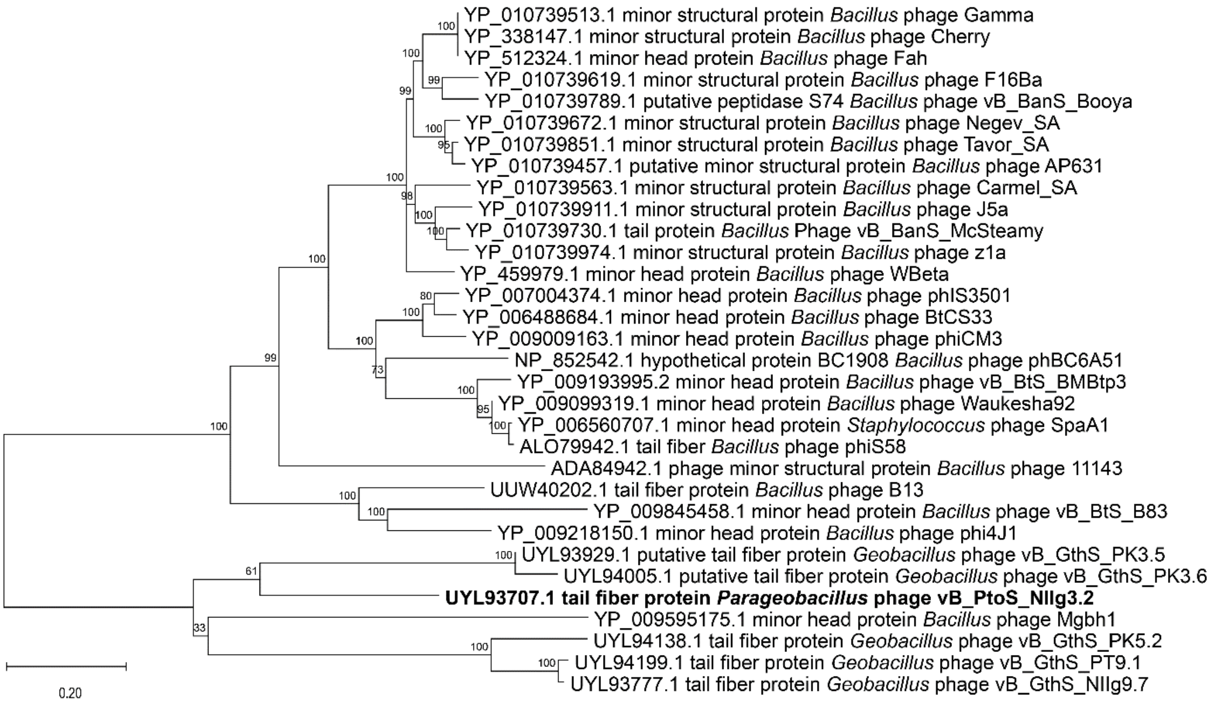

Figure S3. Neighbor-joining tree analysis based on the alignment of the amino acid sequences of *Parageobacillus* bacteriophage NIIg3.2 tail fiber protein (gp17) and its closest BLASTp viral homologues. The phylogenetic analysis was conducted using MEGA version 5. The percentage of replicate trees in which the associated taxa clustered together in the bootstrap test is shown next to the branches.

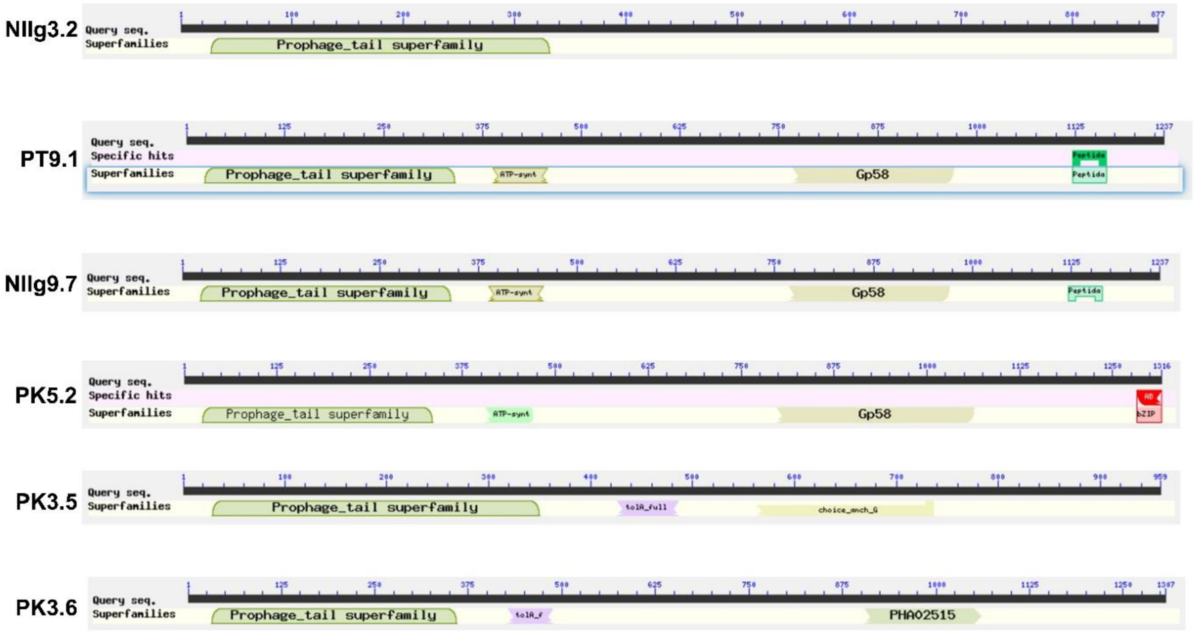

Figure S4. Comparison of conserved domains in tail fiber proteins of thermophilic phages NIIg3.2, PT9.1, NIIg9.7, PK5.2, PK3.5, and PK3.6. Conserved domains were detected using NCBI BLASTp database.

Supplementary references

1. Šimoliūnas, E.; Šimoliūnienė, M.; Laskevičiūtė, G.; Kvederavičiūtė, K.; Skapas, M.; Kaupinis, A.; Valius, M.; Meškys, R.; Kuisienė, N. *Parageobacillus* bacteriophage vB_PtoS_NIIg3.2 – a representative of a new genus within thermophilic siphoviruses. *Viruses*, **2023**, *15*, 1691.
